# Supplementary material for: GapMind: Automated Annotation of Amino Acid Biosynthesis
Source: mSystems. 2020 Jun 23;5(3):e00291-20. doi: 10.1128/mSystems.00291-20 (PMC7311316; doi:10.1128/mSystems.00291-20)
Supplement: TEXT S1 [file mSystems.00291-20-s0001.pdf]

Text S1: Variant pathways and enzymes in GapMind

Besides pathways from MetaCyc, GapMind also describes:

- Proline synthesis via ornithine cyclodeaminase (Graupner and White 2001)
- Arginine synthesis via succinylated intermediates (see the Results)
- Cysteine synthesis via O-succinylserine (Bastard et al. 2017)

Also, GapMind includes tyrosine biosynthesis from phenylalanine (see the Results), even though MetaCyc describes it as occurring only in Metazoa.

GapMind also describes some variant enzymes that might not be expected from MetaCyc's pathway descriptions or from the other curated databases. These variants are from the literature (as opposed to being from our genetic data):

- The methionine synthase from methanogens, which probably uses corrinoid proteins as the methyl donor instead of tetrahydrofolate (Schröder and Thauer 1999).
- The three-part methionine synthase from *Phaeobacter inhibens* (Price et al. 2018).
- The reactivation of vitamin B12 (the reduction of co(II)balamin to co(I)balamin) is described as a separate step that is required for the activity of vitamin B12-dependent methionine synthase. GapMind associates two families with the reactivation of vitamin B12: PF02965, which is part of MetH in *E. coli* and many other bacteria, and the RamA-like domain PF14574 (Ferguson et al. 2009; Price et al. 2018).
- Although MetaCyc includes the reductive sulfuration of aspartate semialdehyde as part of the pathway "L-methionine biosynthesis IV", it links this activity to just one gene. Based on genetic evidence from both *Methanosarcina acetivorans* and *Desulfovibrio vulgaris*, we believe that two other genes are also required (Rauch et al. 2014; Rauch and Perona 2016; Price et al. 2018).
- The histidinol-phosphate phosphatase HisJ from *Bacillus subtilis* (Ghodge et al. 2013). (This protein is annotated in Swiss-Prot, but does not have an experimental evidence code for its catalytic activity, so it was not included in PaperBLAST's database.)
- MetI from *Bacillus subtilis* is O-acetylhomoserine sulfhydrylase and cystathionine γ-synthase (Auger et al. 2002).
- 2-oxobutanoate:ferredoxin oxidoreductase provides an alternate route to 2-oxobutanoate (which is a precursor to isoleucine). MetaCyc describes this reaction but links it to one subunit instead of to both subunits (Fukuda and Wakagi 2002).

References

Auger, S., Yuen, W.H., Danchin, A. and Martin-Verstraete, I. 2002. The metIC operon involved in methionine biosynthesis in Bacillus subtilis is controlled by transcription antitermination. *Microbiology* 148(Pt 2), pp. 507–518.

Bastard, K., Perret, A., Mariage, A., et al. 2017. Parallel evolution of non-homologous isofunctional enzymes in methionine biosynthesis. *Nature Chemical Biology* 13(8), pp. 858–866.

Ferguson, T., Soares, J.A., Lienard, T., Gottschalk, G. and Krzycki, J.A. 2009. RamA, a protein required for reductive activation of corrinoid-dependent methylamine methyltransferase reactions in methanogenic archaea. *The Journal of Biological Chemistry* 284(4), pp. 2285–2295.

Fukuda, E. and Wakagi, T. 2002. Substrate recognition by 2-oxoacid:ferredoxin oxidoreductase from Sulfolobus sp. strain 7. *Biochimica et Biophysica Acta* 1597(1), pp. 74–80.

Ghodge, S.V., Fedorov, A.A., Fedorov, E.V., et al. 2013. Structural and mechanistic characterization of L-histidinol phosphate phosphatase from the polymerase and histidinol phosphatase family of proteins. *Biochemistry* 52(6), pp. 1101–1112.

Graupner, M. and White, R.H. 2001. Methanococcus jannaschii generates L-proline by cyclization of L-ornithine. *Journal of Bacteriology* 183(17), pp. 5203–5205.

Price, M.N., Zane, G.M., Kuehl, J.V., et al. 2018. Filling gaps in bacterial amino acid biosynthesis pathways with high-throughput genetics. *PLoS Genetics* 14(1), p. e1007147.

Rauch, B.J., Gustafson, A. and Perona, J.J. 2014. Novel proteins for homocysteine biosynthesis in anaerobic microorganisms. *Molecular Microbiology* 94(6), pp. 1330–1342.

Rauch, B.J. and Perona, J.J. 2016. Efficient Sulfide Assimilation in Methanosarcina acetivorans Is Mediated by the MA1715 Protein. *Journal of Bacteriology* 198(14), pp. 1974–1983.

Schröder, I. and Thauer, R.K. 1999. Methylcobalamin:homocysteine methyltransferase from Methanobacterium thermoautotrophicum. Identification as the metE gene product. *European Journal of Biochemistry / FEBS* 263(3), pp. 789–796.
