# Supplementary material for: GapMind: Automated Annotation of Amino Acid Biosynthesis
Source: mSystems. 2020 Jun 23;5(3):e00291-20. doi: 10.1128/mSystems.00291-20 (PMC7311316; doi:10.1128/mSystems.00291-20)
Supplement: TEXT S2 [file mSystems.00291-20-s0002.pdf]

Text S2: Potential pathways not included in GapMind

Recent work has suggested that some bacteria can synthesize glycine directly from carbon dioxide and ammonia by using the glycine cleavage system in reverse to convert CO_2, ammonia, 5,10-methylenetetrahydrofolate, and NADH to glycine, tetrahydrofolate, and NAD+ (Figueroa et al. 2018; Tveit et al. 2019). Serine hydroxymethyltransferase (in the reverse of the usual direction) could then form serine. In contrast, most organisms form serine from 3-phosphoglycerate (an intermediate in glycolysis) and use serine hydroxymethyltransferase to obtain glycine. The two bacteria proposed to use the glycine cleavage system in reverse are *Methylocapsa gorgona* MG08 (NCBI assembly GCF_004564215.1) and *Phosphitivorax anaerolimi* Phox-21 (assembly GCA_001896555.1). *M. gorgona* appears to encode the standard serine synthesis pathway, with a high-confidence candidate for all three steps. In *P. anaerolimi*, the presence of serine synthesis from 3-phosphoglycerate is unclear, but it does have candidates for all three steps. Since it remains uncertain if the glycine cleavage system can be the sole source of glycine and serine, these pathways were not included in GapMind.

The MetaCyc entry for L-serine biosynthesis II, which occurs in plants, also suggests that "*Pseudomonas sp.* AM1", which is now known as *Methylorubrum extorquens* AM1, can synthesize serine from glycerate. However the cited paper (Heptinstall and Quayle 1970) actually shows that hydroxypyruvate reductase is required for growth on one-carbon substrates. We now know that this enzyme is part of the serine pathway for assimilating one-carbon units, rather than being dedicated to serine synthesis. So we decided not to include this pathway for serine synthesis (with glycerate as an intermediate) in GapMind.

The MetaCyc pathway L-isoleucine biosynthesis III begins with the glutamate mutase reaction. This pathway is not included in GapMind because glutamate mutase has not been linked to sequence and because no organism has been demonstrated to rely on this pathway to form oxobutanoate (a precursor to isoleucine).

References

Figueroa, I.A., Barnum, T.P., Somasekhar, P.Y., Carlström, C.I., Engelbrektson, A.L. and Coates, J.D. 2018. Metagenomics-guided analysis of microbial chemolithoautotrophic phosphite oxidation yields evidence of a seventh natural CO2 fixation pathway. *Proceedings of the National Academy of Sciences of the United States of America* 115(1), pp. E92–E101.

Heptinstall, J. and Quayle, J.R. 1970. Pathways leading to and from serine during growth of Pseudomonas AM1 on C1 compounds or succinate. *The Biochemical Journal* 117(3), pp. 563–572.

Tveit, A.T., Hestnes, A.G., Robinson, S.L., et al. 2019. Widespread soil bacterium that oxidizes atmospheric methane. *Proceedings of the National Academy of Sciences of the United States of America* 116(17), pp. 8515–8524.
