# Supplementary material for: GapMind: Automated Annotation of Amino Acid Biosynthesis
Source: mSystems. 2020 Jun 23;5(3):e00291-20. doi: 10.1128/mSystems.00291-20 (PMC7311316; doi:10.1128/mSystems.00291-20)
Supplement: TEXT S3 [file mSystems.00291-20-s0003.pdf]

Text S3: Errors in genome sequences or gene models

Six of the gaps in the 35 bacteria with fitness data were due to errors in the genome sequence or to a protein that was not identified, even though an open reading frame is present.

We previously reported a frameshift error in the genome sequence of *Azospirillum brasilense* Sp245 (NCBI assembly GCF_000237365.1) that led to a spurious gap for the histidinol dehydrogenase *hisD* in histidine biosynthesis (Price et al. 2018). Here, we identified two missing gene calls in *A. brasilense*: the phosphoserine transaminase *serC* (over 50% identical to SERC_METBF) and the shikimate kinase *aroL* (over 40% identical to AROK_ECOLI).

In the assembly that we used for *Shewanella oneidensis* MR-1 (NCBI assembly GCF_000146165.1), chorismate synthase *aroC* appears to be truncated or split into two reading frames (SO3078.2 and SO_3079). GapMind classified this as a medium-confidence candidate. Inspection of sequencing data (from TnSeq (Wetmore et al. 2015)) showed that this frameshift is an error in the genome sequence. Also, a proteomics study reported that SO_3079, which is the shorter and downstream part of the protein, is expressed (Romine et al. 2004). This frameshift was corrected in a more recent assembly of *S. oneidensis* (GCF_000146165.2).

Finally, we identified two errors that led to spurious gaps in *Pseudomonas fluorescens* FW300-N1B4 (NCBI assembly GCF_001625455.1). First, by comparing the published assembly to alternate assemblies, we identified a region that was missing; this region included an open reading frame with 84% identity to METZ_PSEAE. Second, an open reading frame with 67% identity to KPRS_ECOLI is present but no protein was predicted. We also identified a frameshift error in the sequence of phosphoribosylanthranilate isomerase that led to a spurious split gene (see Methods).

References

Price, M.N., Zane, G.M., Kuehl, J.V., et al. 2018. Filling gaps in bacterial amino acid biosynthesis pathways with high-throughput genetics. *PLoS Genetics* 14(1), p. e1007147.

Romine, M.F., Elias, D.A., Monroe, M.E., et al. 2004. Validation of Shewanella oneidensis MR-1 small proteins by AMT tag-based proteome analysis. *Omics : a journal of integrative biology* 8(3), pp. 239–254.

Wetmore, K.M., Price, M.N., Waters, R.J., et al. 2015. Rapid quantification of mutant fitness in diverse bacteria by sequencing randomly bar-coded transposons. *mBio* 6(3), pp. e00306-15.
